# The mammalian trafficking chaperone protein UNC93B1 maintains the ER calcium sensor STIM1 in a dimeric state primed for translocation to the ER cortex

**DOI:** 10.1016/j.jbc.2022.101607

**Published:** 2022-01-20

**Authors:** Wen-An Wang, Nicolas Demaurex

**Affiliations:** Department of Cell Physiology and Metabolism, University of Geneva, Geneva, Switzerland

**Keywords:** calcium signaling, innate immunity, ion channels, membrane contact sites, protein trafficking, 3d, triple D, CAD, channel-activating domain, cDNA, complementary DNA, CPA, cyclopiazonic acid, ER, endoplasmic reticulum, HEK-293T, human embryonic kidney-293T cell line, ORMDL3, orosomucoid like 3, PM, plasma membrane, RFP, red fluorescent protein, SAM, sterile alpha motif, SKO, STIM1/2^*−/−*^, SOCE, store-operated Ca^2+^ entry, STING, stimulator of interferon genes, STIM1, stromal interaction molecule 1, SUKO, STIM1/2^*−/−*^ and UNC93B1-deficient cell, Tg, thapsigargin, TIRF, total internal reflection fluorescence, TLR, toll-like receptor, TM, transmembrane, UKO, UNC93B1-deficient cell, UNC93B1, uncoordinated 93 homolog B1, YFP, yellow fluorescent protein

## Abstract

The stromal interaction molecule 1 (STIM1) is an endoplasmic reticulum (ER) Ca^2+^ sensor that regulates the activity of Orai plasma membrane Ca^2+^ channels to mediate the store-operated Ca^2+^ entry pathway essential for immunity. Uncoordinated 93 homolog B1 (UNC93B1) is a multiple membrane-spanning ER protein that acts as a trafficking chaperone by guiding nucleic-acid sensing toll-like receptors to their respective endosomal signaling compartments. We previously showed that UNC93B1 interacts with STIM1 to promote antigen cross-presentation in dendritic cells, but the STIM1 binding site(s) and activation step(s) impacted by this interaction remained unknown. In this study, we show that UNC93B1 interacts with STIM1 in the ER lumen by binding to residues in close proximity to the transmembrane domain. Cysteine crosslinking *in vivo* showed that UNC93B1 binding promotes the zipping of transmembrane and proximal cytosolic helices within resting STIM1 dimers, priming STIM1 for translocation. In addition, we show that UNC93B1 deficiency reduces store-operated Ca^2+^ entry and STIM1–Orai1 interactions and targets STIM1 to lighter ER domains, whereas UNC93B1 expression accelerates the recruitment of STIM1 to cortical ER domains. We conclude that UNC93B1 therefore acts as a trafficking chaperone by maintaining the pool of resting STIM1 proteins in a state primed for activation, enabling their rapid translocation in an extended conformation to cortical ER signaling compartments.

The stromal interaction molecules (STIMs) are endoplasmic reticulum (ER) localized single-pass transmembrane (TM) proteins (1) that mediate the ubiquitous store-operated Ca^2+^ entry (SOCE) signaling pathway. Activated STIM proteins mediate SOCE *via* interactions with plasma membrane (PM) Ca^2+^ permeable channels of the Orai and transient receptor potential families (2, 3, 4). The predominant isoform STIM1 and its homolog STIM2 are activated by Ca^2+^ depletion of the ER with distinct kinetics, as STIM1 has a Ca^2+^-binding *K*_*d*_ of ∼200 μM (5) and is therefore less sensitive to store depletion than STIM2, which has a *K*_*d*_ of ∼500 μM (6). Functional and structural studies have focused on STIM1 and have elucidated the sequential conformational changes that occur during its activation. Ligand binding to PM or intracellular receptors coupled to phospholipase C leads to the production of inositol triphosphate, which activates receptors located on the ER membrane to cause ER Ca^2+^ release (5, 7). The Ca^2+^ depletion of the ER is sensed by STIM1 *via* the release of Ca^2+^ from its EF-hand and sterile alpha motif (SAM) domains located within the ER lumen, leading to the apposition and dimerization of the TM region. The conformational change is transmitted to the cytosolic domain into the extension of the proximal coiled-coil (CC1α1) domain, which allows the release of the channel-activating domain (CAD), also known as the STIM1/Orai1 activating region, into an elongated conformation (8). The elongation of STIM1 oligomers exposes a polybasic C-terminal tail that binds to negatively charged lipids, allowing STIM1 to accumulate in cortical ER domains tethered to the PM (5, 8, 9, 10, 11). At these ER–PM junctions, STIM1 can trap and gate Orai1 Ca^2+^ channels, eliciting an influx of Ca^2+^ from the extracellular milieu into the cytoplasm. The influx of Ca^2+^ sustains Ca^2+^-dependent signaling events and provides a source of Ca^2+^ for the replenishment of ER Ca^2+^ stores *via* the sarco/ER Ca^2+^-ATPase pump (12, 13, 14, 15, 16, 17).

SOCE regulates signaling pathways that control gene expression, protein secretion, cell growth, proliferation and apoptosis, tissue differentiation, and organ development in most species and cell types. In humans, SOCE is critically important for a variety of immune responses, including T-cell proliferation (18, 19, 20, 21), platelet activation (22, 23), muscle differentiation and tonicity (24), and antigen cross-presentation by dendritic cells (25). Given the plethora of cellular functions that rely on SOCE, STIM and Orai proteins have numerous binding partners that regulate their activation and termination steps. Among STIM1 binding partners, we discovered that the uncoordinated 93 homolog B1 (UNC93B1) controls the activation of STIM1 to regulate antigen cross-presentation in dendritic cells (26).

The mammalian *UNC93B1* is a highly conserved gene that is related to *unc-93*, a *Caenorhabditis elegans* gene that encodes a subunit of a two-pore potassium channel involved in the coordination of muscle contraction (27, 28). UNC93B1 has been characterized as a multiple membrane-spanning protein that localizes to the ER and is predicted to have 12 TM domains with the C-terminal and N-terminal regions facing the cytoplasm (29). Mammalian UNC93B1 has only 18% sequence identity with its *C. elegans* homolog and was shown in the last decade to play an important and entirely different function (30). The identification and characterization of herpes simplex virus-1 encephalitis in two children with autosomal recessive deficiency of UNC93B1 revealed a link between UNC93B1 and antiviral immunity (31). In mice, a *UNC93B1* “triple D” (3d) mutation (characterized as a single amino acid substitution, H412R, in the ninth TM domain) impairs signaling from toll-like receptors (TLRs) 3, 7, and 9, leading to defective antigen cross-presentation *via* the major histocompatibility complex class I and exogenous antigen presentation *via* major histocompatibility complex class II (30). Subsequent studies showed that UNC93B1 functions as an ER protein chaperone and that its interaction with TLRs 3, 5, 7, 8, and 9 are crucial for a proper TLR localization and signaling during innate immune responses against foreign microbes and viruses (32, 33, 34). Recent cryo-EM structures of TLR3–UNC93B1 and TLR7–UNC93B1 complexes indicate that both TLRs interact with UNC93B1 *via* their TM and luminal juxtamembrane regions, with different oligomerization states (35). Recently, UNC93B1 was shown to interact and traffic along with the stimulator of interferon genes (STING) and to negatively regulate innate immune responses to cytosolic DNA stimulation and DNA virus infection (36). STIM1 was also reported to interact with STING and prevent its translocation to the ER–Golgi intermediate compartment, an interaction that limits the expression of type I interferons after viral infections (37). These studies implicate both UNC93B1 and STIM1 in the trafficking of ER signaling proteins and highlight the importance of the interactions between UNC93B1 and STIM1 in antiviral immunity.

In this study, we show that UNC93B1 interacts with STIM1 in nonimmune cells and characterize the biochemical and functional impacts of this molecular interaction. We show that UNC93B1 facilitates the formation of dimers within STIM1 TM and proximal cytosolic helixes. UNC93B1 gatekeeping function maintains STIM1 in a dimeric state primed for translocation to cortical ER domains during SOCE.

## Results

### Unc93B1 deficiency reduces SOCE in human embryonic kidney-293T cells

**Figure 1:**
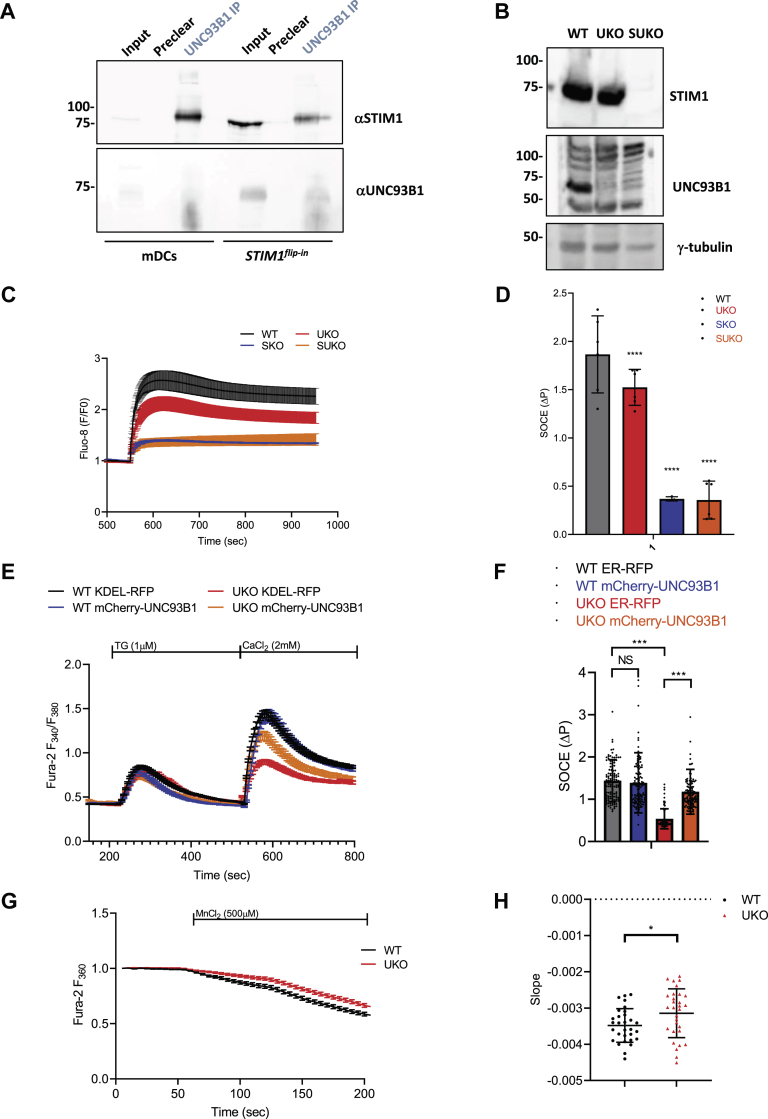
**UNC93B1 deficiency reduces store-operated Ca**^**2+**^**entry in HEK-293T cells.***A*, Western blot of protein lysates from mouse dendritic cells (mDCs) and HEK-293T *STIM1*^*flip-in*^ cells immunoprecipitated with αUNC93B1 antibody and probed with αSTIM1 to detect STIM1-UNC93B1 interaction (*top*) and αUNC93B1 to detect UNC93B1 precipitation (*bottom*). *B*, Western blot of protein lysates from WT, *UNC93B1*^*−/−*^ (clone 52, UKO), and *STIM1/2*^*−/−*^/*UNC93B1*^*−/−*^ (clone 30, SUKO) HEK-293T cells probed with αSTIM1, αUNC93B1, and αγ-tubulin antibodies. *C*, averaged Fluo-8 responses evoked by Ca^2+^ readmission to 1 μM Tg-treated WT, SKO, UKO, and SUKO cells. *D*, peak amplitude of the responses in *C*. Data are mean ± SD from two independent experiments in triplicate. ∗∗∗∗*p* > 0.001, ordinary one-way ANOVA (*p* < 0.0001, *F* = 46.20). *E*, averaged Fura-2 responses evoked by 1 μM Tg and subsequent Ca^2+^ readmission to WT and UKO cells expressing KDEL-RFP or mCherry-UNC93B1. *F*, peak amplitude of the SOCE responses in *E*. Data are mean ± SD of 120 cells from three independent experiments. NS; ∗∗∗*p* > 0.005, ordinary one-way ANOVA (*p* < 0.0001, *F* = 71.67). *G*, averaged recordings of Fura-2 fluorescence quenching at the isosbestic wavelength (360 nm) during addition of MnCl_2_ (500 μM) to untreated WT and UKO cells. *H*, statistical evaluation of the Mn^2+^-induced quench rates. Data are mean ± SD of 123 cells from three independent experiments. ∗*p* > 0.05, unpaired *t* test. HEK-293T, human embryonic kidney 293T cell; NS, nonsignificant; RFP, red fluorescent protein; SOCE, store-operated Ca^2+^ entry; STIM1, stromal interaction molecule 1; Tg, thapsigargin.

UNC93B1 is a protein chaperone for TLRs that interacts with STIM1 to positively regulate SOCE in dendritic cells (26). Like STIM1, UNC93B1 is ubiquitously expressed according to the Human Protein Atlas (38), suggesting that its chaperone function might extend beyond immune cells. To test this possibility, we assessed whether UNC93B1 binds to STIM1 and regulates Ca^2+^ fluxes in human embryonic kidney-293T (HEK-293T) cells that lack expression of TLR1–10 (39). A ∼70 kDa band was detected by Western blot with an anti-UNC93B1 antibody, indicating that UNC93B1 is expressed in HEK-293T cells (Figs. 1*B* and S1*A*). The UNC93B1 immunoreactivity was not altered by the genetic ablation of the two STIM isoforms but was enhanced by the stable overexpression of STIM1 with the flip-in system (Fig. S1*A*). We could observe an interaction between STIM1 and UNC91B1 in a mouse dendritic cell line and in these HEK-293T cells (Fig. 1*A*), confirming that the two proteins interact regardless of TLR expression, in both immune and nonimmune cell types. To further study nonimmunological cell lines, we then disrupted the endogenous *UNC93B1* gene by CRISPR in WT or *STIM1/2*^*−/−*^ (SKO) HEK-293T cells and generated *UNC93B1*-deficient (UKO) or *STIM1/2*^*−/−*^ and UNC93B1-deficient (SUKO) cells (Fig. S1*B*). Lack of UNC93B1 protein expression was validated by Western blot (Fig. 1*B*). STIM1 protein and mRNA levels were identical in WT and UKO cells (Figs. 1*B* and S2*A*) as were the rates of STIM1 protein degradation following treatment with cycloheximide (Fig. S2, *B* and *C*). These data indicate that the STIM1–UNC93B1 interaction occurs beyond TLR-specialized cells and that the synthesis and degradation of the endogenous STIM1 protein is not affected by UNC93B1 deficiency.

We then assessed the functional impact of UNC93B1 deficiency on STIM1 activity, using thapsigargin (Tg) to induce ER Ca^2+^ store depletion and evoke SOCE. SOCE was significantly decreased in UKO cells and abrogated in SKO and SUKO cells when measured with Fluo-8 on a plate reader (Fig. 1, *C* and *D*). Fura-2 microfluorimetric recordings confirmed that UKO cells had reduced SOCE, which was restored to control levels by the re-expression of mCherry-UNC93B1, thereby linking the SOCE defect to UNC93B1 deficiency (Fig. 1, *E* and *F*). We then used the reversible sarco/ER Ca^2+^-ATPase inhibitor cyclopiazonic acid (CPA) to perform two successive rounds of Ca^2+^ release and readmission. The amplitude of the first SOCE component as well as the amount of Ca^2+^ mobilized by the second addition of CPA were reduced in UNC93B1-deficient cells, confirming the SOCE defect (Fig. S3). Interestingly, the amplitude of the second SOCE component was comparable in control and UNC93B1-deficient cells (Fig. S3, *A* and *B*), suggesting that the SOCE defect linked to UNC93B1 deficiency disappears during this longer protocol of repetitive store depletion.

To assess whether UNC93B1 deficiency alters the basal activity of Ca^2+^ entry channels, we measured the rates of fura-2 fluorescence quenching by Mn^2+^, a divalent cation that permeates Ca^2+^ channels. In cells not previously exposed to Tg, the Mn^2+^ quench rates were reduced in UKO cells, indicating a reduced basal Ca^2+^ channel activity (Fig. 1, *G* and *H*). UNC93B1 deficiency thus reduces basal and SOCE.

### Effect of UNC93B1 deficiency on STIM1 trafficking and OraI1 gating

**Figure 2:**
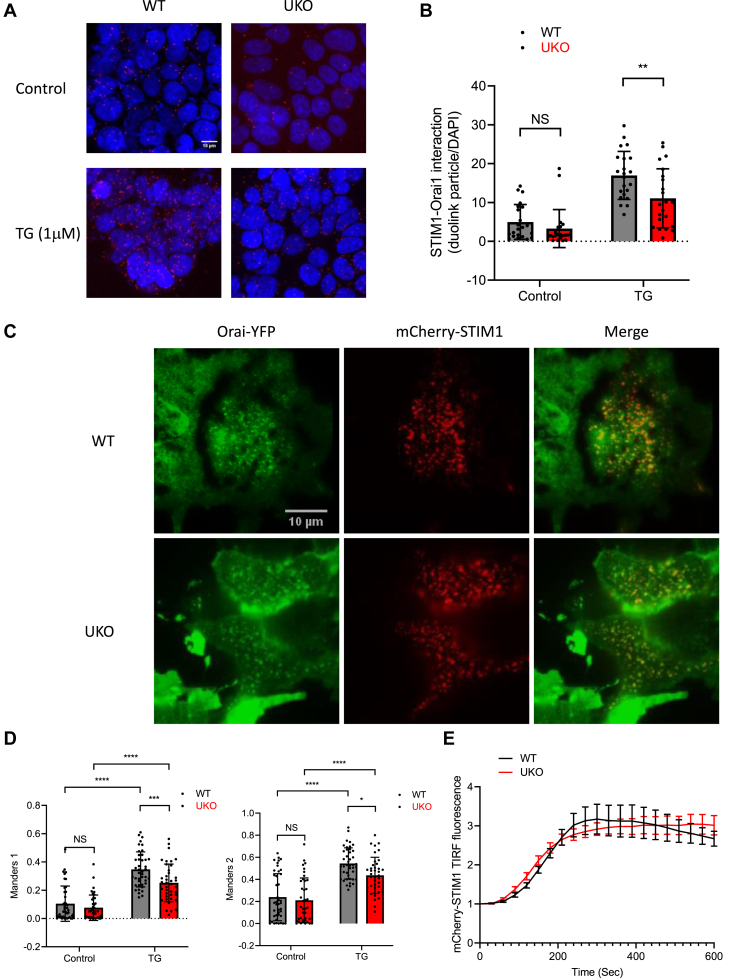
**UNC93B1 deficiency reduces STIM1–ORAI1 interactions.***A*, representative confocal images of WT and UKO cells stained with DAPI (*blue*) and the Duolink proximity ligation assay using mouse αSTIM1 and rabbit αORAI1 antibodies (*red*) before and 10 min after 1 μM Tg addition. *B*, numbers of immunoreactive dots per cell revealed by the proximity ligation assay. Data are mean ± SD of 22 images from six samples per condition. NS; ∗∗*p* > 0.01, two-way ANOVA (interaction: SS = 96.70, DF = 1, MS = 96.70, *F*(1,84) = 2.758, *p* = 0.1005). *C*, representative TIRF images of WT and UKO cells expressing Orai1-YFP (*green*) and mCherry-STIM1 (*red*) treated with 1 μM Tg for 10 min. *D*, Manders 1 and 2 colocalization index between Orai1-YFP and mCherry-STIM1 before and after Tg addition. Data are mean ± SD of 36 images from three independent experiments. NS; ∗∗∗*p* > 0.005; ∗∗∗∗*p* > 0.001, two-way ANOVA (M1 interaction: SS = 0.04521, DF = 1, MS = 0.04521, *F*(1,79) = 7.136, *p* = 0.0092; M2 interaction: SS = 0.06389, DF = 1, MS = 0.06389, *F*(1,79) = 5.191, *p* = 0.0254). *E*, time course of Tg-induced changes in mCherry-STIM1 TIRF fluorescence in WT and UKO cells. DAPI, 4′,6-diamidino-2-phenylindole; NS, nonsignificant; STIM1, stromal interaction molecule 1; Tg, thapsigargin; TIRF, total internal reflection fluorescence; UKO, *UNC93B1*-deficient cell.

To investigate the underlying mechanism, we quantified the impact of UNC93B1 deficiency on the STIM1–Orai1 interactions occurring in resting cells and during store depletion. Store depletion exposes the STIM1 CAD domain that mediates the trapping and gating of Orai1 channels in PM clusters (40, 41). Accordingly, the interactions between endogenous STIM1 and Orai1, measured with a proximal ligation assay, increased by approximately threefold after addition of Tg (Fig. 2, *A* and *B*). In UKO cells, the STIM1–Orai1 interactions were slightly reduced at rest and significantly reduced after Tg, compared with WT cells (Fig. 2, *A* and *B*). Reduced interactions between mCherry-STIM1 and Orai1-yellow fluorescent protein (YFP) were also observed in UKO cells by total internal reflection fluorescence (TIRF) microscopy, the colocalization coefficients being lower at rest (Figs. S4*A* and 2*D*) and significantly smaller in UKO than in WT cells after Tg addition (Fig. 2, *C* and *D*). UNC93B1 deficiency therefore reduces the interactions between endogenous and exogenous STIM1 and Orai1 proteins.

**Figure 3:**
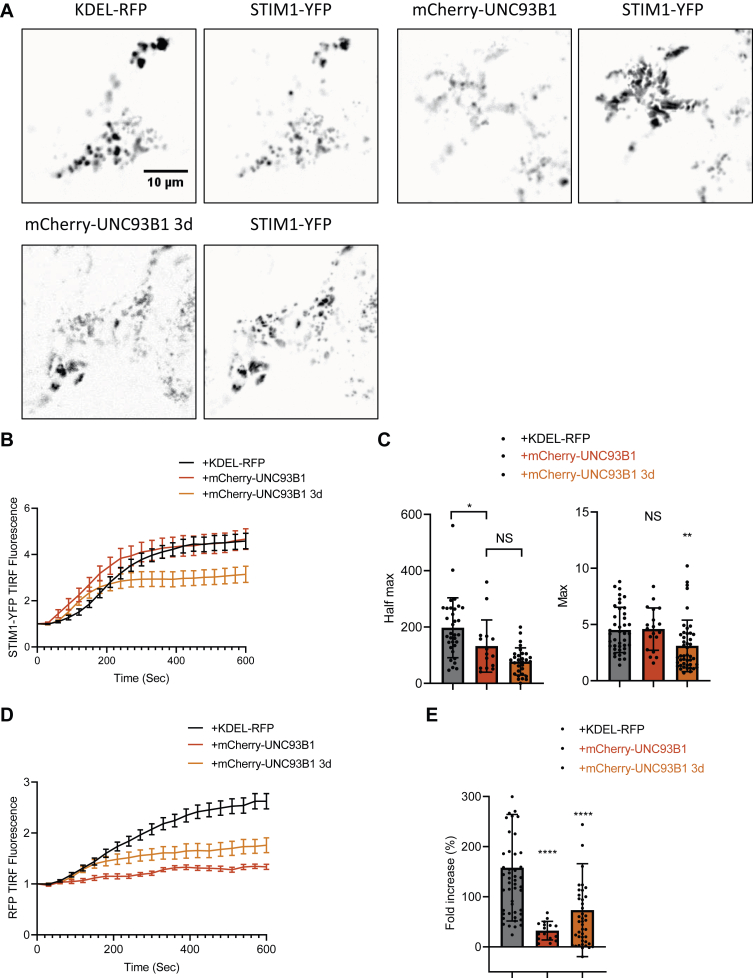
**UNC93B1 facilitates STIM1 translocation without reaching ER–PM junctions.***A*, representative TIRF images of WT cells expressing STIM1-YFP (*right images*) together with KDEL-RFP, mCherry-UNC93B1, or mCherry-UNC93B1 3d (*left images*) after exposure to 1 μM Tg for 10 min to induce STIM1-YFP clustering. *B*, time course of 1 μM Tg-induced changes in STIM1-YFP TIRF fluorescence. *C*, kinetics (*left*) and amplitude (*right*) of the changes in STIM1-YFP fluorescence. Data are mean ± SD of 26 to 69 cells from four independent experiments. NS; ∗*p* > 0.05; ∗∗*p* > 0.01, ordinary one-way ANOVA (half-maximum *F* = 15.42, *p* < 0.0001; maximum *F* = 5.915, *p* = 0.0037). *D*, time course of Tg-induced changes in KDEL-RFP, mCherry-UNC93B1, and mCherry-UNC93B1 3d fluorescence. *E*, averaged amplitude of the changes in RFP or mCherry fluorescence. Data are mean ± SD of 26 to 69 cells from four independent experiments. ∗∗∗∗*p* > 0.001, ordinary one-way ANOVA (*F* = 15.61, *p* < 0.0001). ER, endoplasmic reticulum; NS, nonsignificant; PM, plasma membrane; RFP, red fluorescent protein; STIM1, stromal interaction molecule 1; Tg, thapsigargin; TIRF, total internal reflection fluorescence.

Interestingly, UNC93B1 ablation did not impact the Tg-induced increase in TIRF fluorescence of mCherry-STIM1 coexpressed with Orai1-YFP (Fig. 2*E*). This indicates that the reduced STIM1–Orai1 interactions are not because of reduced recruitment of mCherry-STIM1 to the TIRF plane as was previously reported for the dominant-negative UNC93B1 “3d” mutant (26) but instead reflect a reduced ability of mCherry-STIM1 activated in the absence of UNC93B1 to trap Orai1 channels at the PM. To establish this point, we measured the kinetics of mCherry-UNC93B1 and STIM1-YFP cotranslocation to the PM by TIRF imaging, using the “3d” UNC93B1 mutant and the ER marker red fluorescent protein (RFP)-KDEL as controls. Enrichment of the luminal RFP-KDEL protein in ER–PM contact sites provides reference values to quantify the relative Tg-induced enrichment or exclusion of other proteins bearing similar tags (42). Cells expressing the constructs at similar levels were selected based on their YFP and RFP/mCherry fluorescence (Fig. S4, *B* and *C*). Following Tg addition, STIM1-YFP accumulated in the TIRF plane along with KDEL-RFP (Fig. 3*A*). The kinetics of STIM1-YFP recruitment were accelerated by the coexpression of WT and “3d” mCherry-UNC93B1, the time to reach half-maximal fluorescence intensity decreasing by more than 1 min (Fig. 3, *B* and *C*). The extent of STIM1-YFP accumulation was not affected by the expression of WT UNC93B1 but was reduced by the expression of the “3d” mutant, consistent with earlier reports (Fig. 3*C*). A reduced amount of STIM1-YFP thus reached the TIRF plane faster when coexpressed with UNC93B1-3D, possibly as overexpressed STIM1-YFP monomers bypassing the molecular step(s) impacted by the “3d" mutant. Interestingly, mCherry-UNC93B1 was not detected in the TIRF plane following Tg addition, whereas partial accumulation of the “3d” mutant was observed (Fig. 3, *D* and *E*). These data indicate that UNC93B1 accelerates the recruitment of STIM1 to cortical ER domains without populating these structures itself, and that the translocation process is antagonized by the dominant-negative UNC93B1 “3d” mutant.

**Figure 4:**
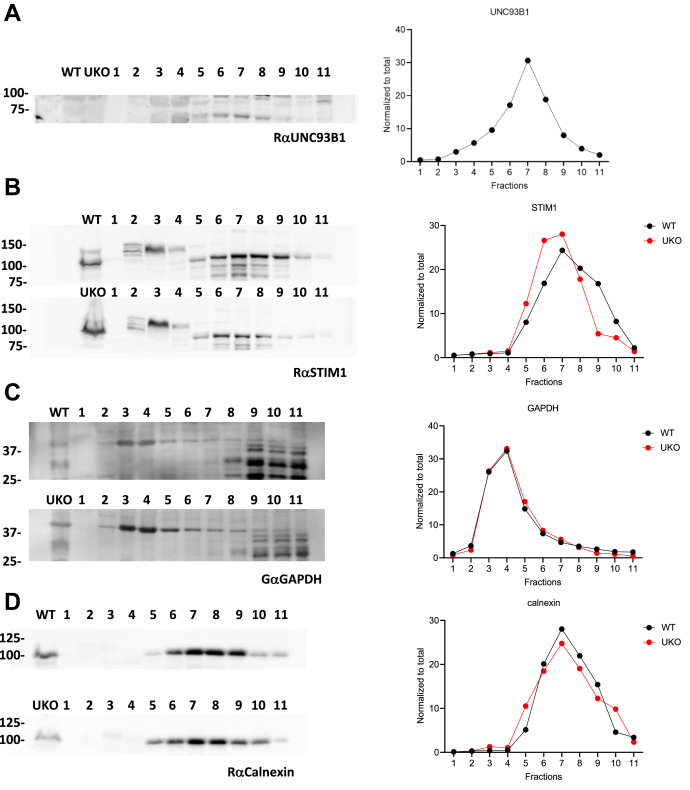
**UNC93B1 deficiency shifts STIM1 to lighter ER domains.***A*, *left*, immunoblot of protein lysates of WT cells subjected to subcellular fractionation (1–11; *light* to *heavy*) through an iodixanol gradient (6%, 9%, 12%, 15%, 18%, 21%, 25%, and 27%), UKO cell lysate is a negative control, probed with αUNC93B1. *Right*, quantification of UNC93B1 immunoreactivity through fractions 1 to 11. *B*–*D*, *left*, immunoblots of subcellular fractions from WT (*top*) and UKO (*bottom*) cells probed with αSTIM1 (*B*), αGAPDH (*C*), and αCalnexin (*D*) antibodies. *Right*, quantification of STIM1 (*B*), GAPDH (*C*), and calnexin (*D*) immunoreactivity through fractions 1 to 11. Representative of two biological experiments. ER, endoplasmic reticulum; STIM1, stromal interaction molecule 1; UKO, *UNC93B1*-deficient cell.

The observation that UNC93B1 alters STIM1 mobility without itself translocating to the PM suggested that the binding occurs in a perinuclear ER compartment. We then attempted to establish the subcellular ER distribution of UNC93B1 and STIM1 biochemically. We subjected cellular membranes from WT and UKO cells to centrifugation over an iodixanol gradient to separate organelles based on density. In WT cells, both UNC93B1 and STIM1 accumulated in heavy fractions positive for ER marker calnexin (fractions 5–10) clearly separated from the lighter cytoplasmic GAPDH-positive fractions (fractions 2–6) (Fig. 4). The lighter fractions 2 to 3 contained a small amount of late endosome marker Rab7 and fractions 3 to 4 sphingolipid biosynthesis regulator 3 (orosomucoid like 3 [ORMDL3]) (Fig. S5). The heavier fractions 5 to 9 contained the PM marker plasma membrane Ca2+-ATPase, the majority of Rab7, and fractions 5 to 8 the lysosomal marker lysosomal-associated membrane protein 1 (Fig. S5). The distribution of these markers was not different between WT and UKO cells (Figs. 4 and S5), but the distribution of STIM1 was shifted from the heavier fraction 9 to the lighter fraction 6 in the absence of UNC93B1 (Fig. 4*B*). This mobility shift suggests that UNC93B1 deficiency promotes the accumulation of STIM1 in specific ER domains of distinct biochemical composition.

### UNC93B1 binds to the proximal region of STIM1 luminal domain

**Figure 5:**
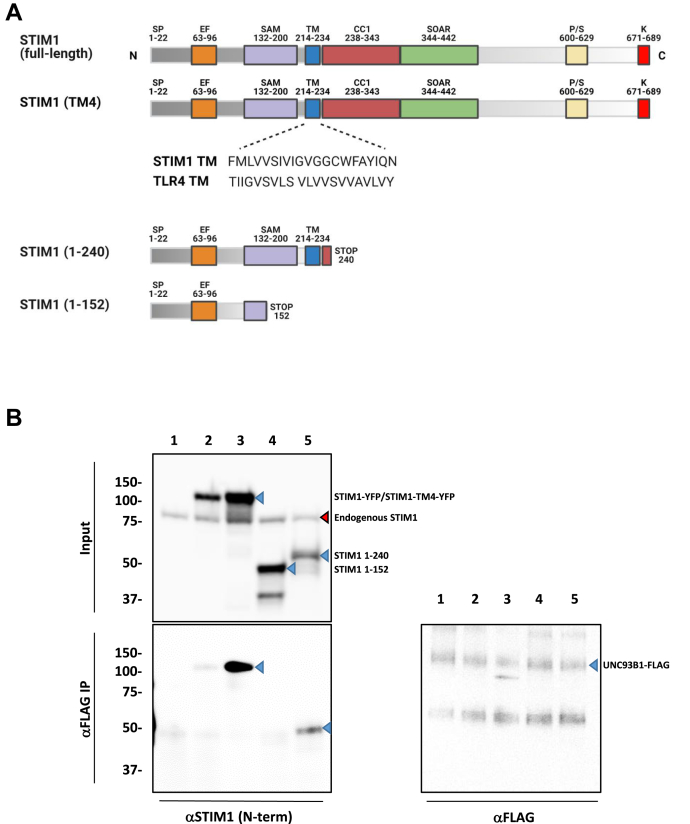
**UNC93B1 binds to the luminal juxtamembrane region of STIM1.***A*, scheme showing the relevant domains within STIM1 full-length, STIM1^TM4^ (TM domain swapped for TLR4 TM domain), STIM1^1–240^, and STIM1^1–152^. *B*, immunoblot of input and immunoprecipitated (IP) protein lysates from WT cells expressing UNC93B1-FLAG with KDEL-GFP (1), STIM1-YFP (2), STIM1^TM4^-YFP (3), mCherry-STIM1^1–152^ (4), or mCherry-STIM1^1–240^ (5). UNC93B1 was immunoprecipitated with αFLAG antibody, and the blots were probed with αSTIM1 (N-term) and αFLAG antibodies. The *blue arrows* indicate the locations of the expected products, and the *red arrows* indicate the additional reactivities. Representative of two biological experiments. STIM1, stromal interaction molecule 1; TLR4, toll-like receptor 4; TM, transmembrane; YFP, yellow fluorescent protein.

We previously showed that UNC93B1 interacts with STIM1 constructs lacking the cytoplasmic domains (26), implying that the binding occurs within the ER luminal or TM domain of STIM1. Moreover, UNC93B1 does not interact with STIM1 D76A (26), a constitutively active mutant prerecruited to ER–PM contact sites (43, 44), indicating that UNC93B1 preferentially binds to the resting conformation of STIM1. We then attempted to identify the STIM1 region mediating its interaction with UNC93B1. UNC93B1 interacts with TLR3, TLR7, and TLR9 but not with TLR4 or with a TLR9 chimera bearing the TLR4 TM domain (32), suggesting that UNC93B1 might bind its targets *via* their TM domain. To test whether UNC93B1 binds to STIM1 TM domain, we generated a STIM1 chimera containing the TLR4 TM domain (STIM1^TM4^) and assessed the interaction by coimmunoprecipitation (Fig. 5*A*). STIM1^TM4^ restored SOCE in SKO cells, indicating that the TM chimera is functional (Fig. S6*A*). To our surprise, STIM1^TM4^ protein coimmunoprecipitated with UNC93B1 (Fig. 5*B*), indicating that the TM domain of STIM1 is dispensable for the interaction. We then assessed the binding of UNC93B1 to STIM1 truncation mutants containing the entire luminal domain, STIM1^1–240^, or a luminal segment lacking the juxtamembrane domain, STIM1^1–152^ (Fig. 5*A*). STIM1^1–240^ but not STIM1^1–152^ coimmunoprecipitated with UNC93B1 (Figs. 5*B* and S7). We then tested the functional effect of these two truncation mutants. Expression of STIM1^1–240^, which interacts with UNC93B1, significantly reduced SOCE in WT but not in UNC93B1-deficient cells, whereas the binding-deficient STIM1^1–152^ mutant did not impact SOCE in either cell type (Fig. S6, *B*–*D*). These data indicate that the binding site for UNC93B1 lies within the ER lumen between amino acids 152 and 214, a region distal to the STIM EF hands and encompassing part of the SAM domain. Moreover, the STIM1 truncation mutant that interacts with UNC93B1 exerts a dominant-negative effect that requires the presence of UNC93B1.

### Effect of UNC93B1 deficiency on STIM1 activation steps

**Figure 6:**
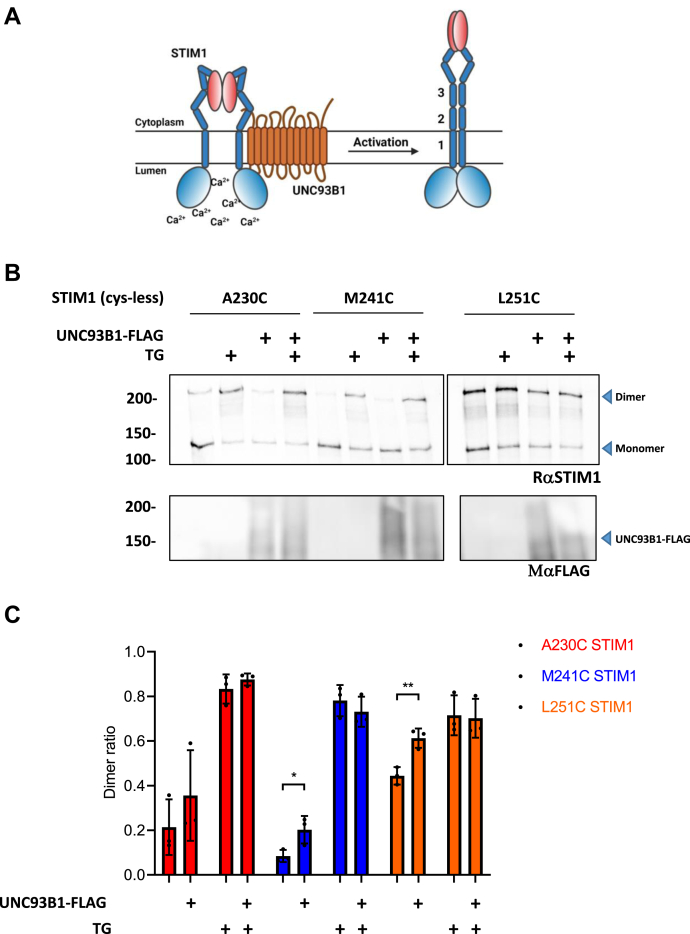
**UNC93B1 increases the formation of resting STIM1 dimers.***A*, scheme showing the STIM1 activation steps tested in the cross-linking assay. STIM1 activation initiated by the unbinding of Ca^2+^ ions from luminal EF-hand domains induces the sequential apposition of STIM1 TM and proximal cytosolic helixes. This zipping mechanism can be revealed by crosslinking cysteine-less STIM1 constructs bearing a single cysteine positioned in the TM domain (1-A230C), proximal cytosolic helix (2-M241C), or second cytosolic helix (3-L251C). *B*, immunoblot analysis of protein lysates from SUKO cells expressing the single cysteine STIM1 constructs with or without UNC93B1-FLAG and treated or not with Tg (0.5 μM for 5 min). Cells were subjected to *in vivo* crosslinking with aldrithiol-4 and to electrophoresis under nonreducing conditions. Blots were probed with αSTIM1 and αFLAG antibodies. Representative of three independent experiments. *C*, quantification of the STIM1 dimer ratio from the *in vivo* crosslink immunoblots. Data are mean ± SD of three experiments. Nonsignificant where there is no indication, ∗*p* < 0.05; ∗∗*p* < 0.01, unpaired *t* test. STIM1, stromal interaction molecule 1; Tg, thapsigargin; TM, transmembrane.

SOCE is initiated by the release of Ca^2+^ ions from the EF-SAM domain of STIM1, which causes the dimerization of the EF-SAM region, the TM helices, and the juxtamembrane domains in an extended coiled-coil confirmation that releases a cytoplasmic intramolecular clamp and exposes the CAD (8, 45). Since the UNC93B1 “3d” mutation impairs STIM1 oligomerization (26), we tested whether the separable dimerization steps of STIM1 activation were affected by UNC93B1 deficiency. To track the apposition of specific STIM1 domains, we used cysteine-less STIM1 mutants reconstituted with a single cysteine at residue 230 (TM region), 241 (juxtamembrane region), or 251 (CC1 domain) (Fig. 6*A*). These STIM1 constructs were shown to form differing levels of *in vitro* cross-linking dimers in the presence and absence of Ca^2+^ (8). To test whether UNC93B1 facilitates the formation of intramolecular dimers between TM helices and/or proximal CC1 regions, we expressed these STIM1 cysteine mutants with or without UNC93B1-FLAG in our SUKO cells and conducted *in vivo* crosslinking in the presence and absence of Tg. UNC93B1-FLAG significantly increased the proportion of cross-linked dimers in resting cells expressing the M241C and L251C cysteine mutants, whereas in the presence of Tg, the dimer ratio was maximal in all conditions (Fig. 6*C*). Furthermore, mCherry-UNC93B1-3d did not promote the formation of cross-linked dimers between any of the cysteine mutants tested (Fig. S8). These data indicate that WT but not UNC93B1-3d facilitates the formation of STIM1 dimers in resting cells by acting at an early step before or during the apposition of STIM1 TM helices.

## Discussion

In this study, we report that the mammalian UNC93B1 binds to the juxtamembrane region of STIM1 luminal domain to regulate STIM1 trafficking and function. UNC93B1 is a trafficking chaperone of nucleic acid–sensing TLR that plays an essential role in innate immunity by restricting the activation of these TM receptors to endolysosomal compartments (46). UNC93B1 was also recently shown to target the signaling adaptor STING for degradation (36), whereas STIM1 was shown to retain STING in the ER (37). We previously showed that interactions between UNC93B1 and STIM1 facilitate the crosspresentation of antigens by dendritic cells (26). We now extend these observations to nonimmune cells by showing that UNC93B1 is expressed and interacts with STIM1 in HEK-293T cells. We further show that UNC93B1 does not require the TM domain of STIM1 for protein–protein interaction and restrict the necessary interaction site to residues 152 to 214 within the STIM1 luminal domain, a region distal to STIM1 EF hands and encompassing part of the SAM domain. UNC93B1 therefore binds to a luminal region within STIM1 that experiences the initial conformational change during ER Ca^2+^ depletion.

Using Ca^2+^ assays and TIRF imaging, we show that UNC93B1 deficiency reduces SOCE in HEK-293T cells and that this defect is associated with reduced interactions between endogenous STIM1 and Orai1 proteins. Since Orai1 trapping and gating by STIM1 is the molecular step that triggers Ca^2+^ entry, the reduced SOCE most likely derives from the reduction in STIM–Orai1 interactions. A repeated stimulation restored SOCE to control levels, indicating that productive interactions could be generated by providing additional time for STIM1 to trap enough Orai1 channels. Interestingly, basal Ca^2+^ entry and STIM1–Orai1 interactions were also reduced in UNC93B1-deficient cells prior to Tg addition, suggesting that the functional defect associated with UNC93B1 deficiency is already detectable in naïve store-replete cells. Accordingly, we show by subcellular fractionation that UNC93B1 deficiency shifts STIM1 to lighter ER subcellular fractions in unstimulated cells. The significance of this STIM1 shift to lighter ER fractions requires further investigation linking the density of subcellular fractions to a specific ER location at contact sites or to a Ca^2+^ storage function. However, this observation does confirm that the consequence of the lack of UNC93B1 is already noticeable in naïve cells and suggests that UNC93B1 acts as an intracellular trafficking chaperone for STIM1 to control its location within specific ER subdomains.

**Figure 7:**
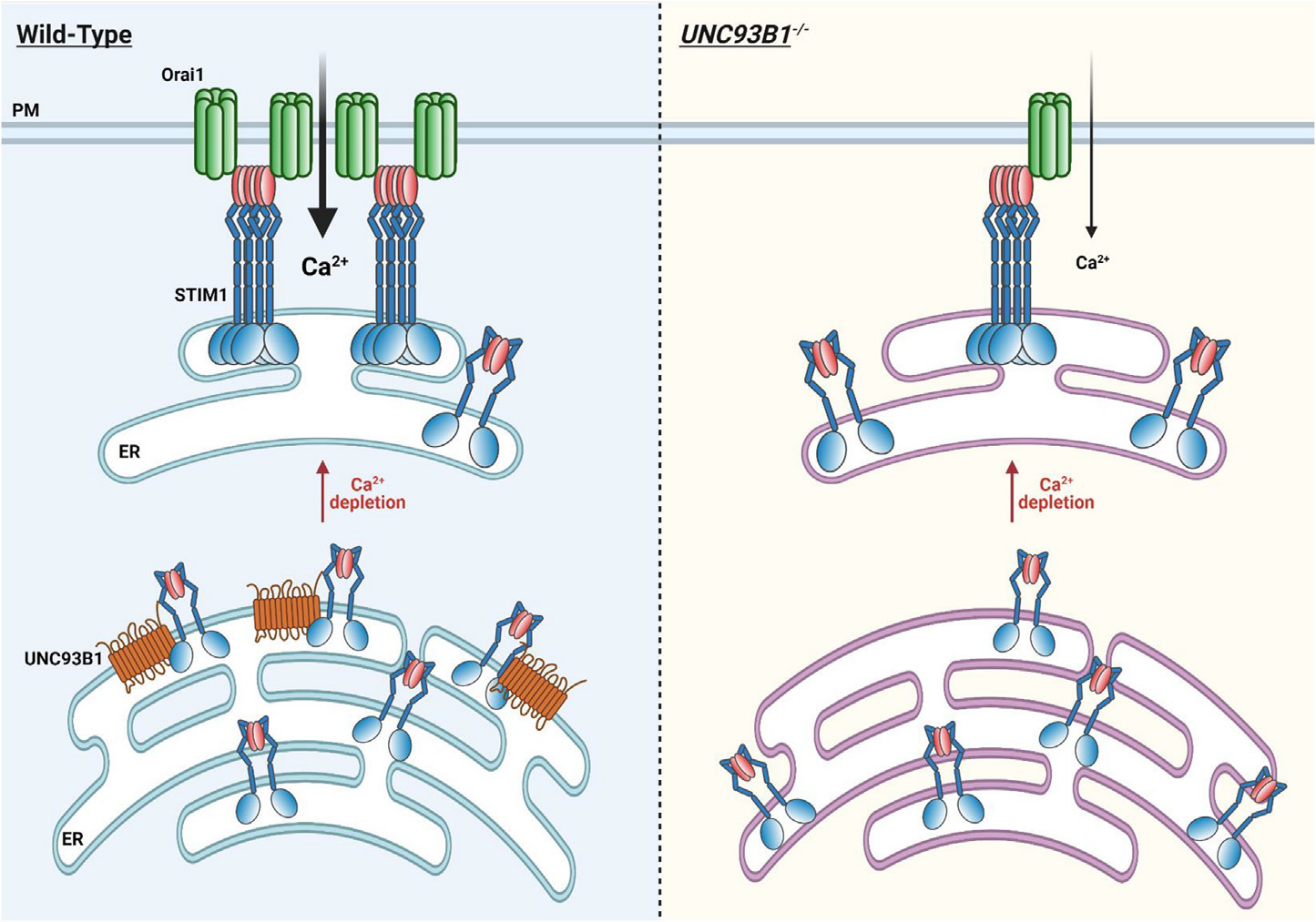
**Graphical model.** Graphical representation of the role of UNC93B1 as a chaperone that facilitates the early steps of STIM1 activation following ER Ca^2+^ depletion. UNC93B1 binds to STIM1 at rest and promotes the initial conformational step of its activation cascade, namely the zipping of the TM and proximal coiled-coil domains (*dark blue bars*) that cause STIM1 to adopt an extended conformation with an exposed channel-activating domain (*red ovals*) that efficiently traps and activates Orai1 channels (*green*) at the PM (WT, *left panel*). In the absence of UNC93B1, the maintenance of optimal STIM1–Orai1 coupling is lost (*UNC93B1*^*−/−*^, *right panel*). ER, endoplasmic reticulum; PM, plasma membrane; STIM1, stromal interaction molecule 1; TM, transmembrane.

Unexpectedly, UNC93B1 deficiency reduced STIM1–Orai1 interactions without preventing the recruitment of STIM1-YFP to the TIRF plane. This indicates that STIM1 can reach cortical ER domains when overexpressed in the absence of UNC93B1 but in a conformation that does not allow the efficient trapping of Orai1 channels. Coexpression of mCherry-UNC93B1 accelerated STIM1-YFP recruitment, indicating that chaperoning by UNC93B1 impacts STIM1 mobility, an effect also observed with mCherry-UNC93B1-3d despite the reduced amounts of STIM1-YFP reaching the TIRF plane. UNC93B1-3d interferes with an early step of STIM1 oligomerization to limit the amounts of STIM1-YFP oligomers reaching the PM (26), and the faster apparent recruitment likely reflects the transit of inactive STIM1-YFP monomers bypassing the molecular step(s) prevented by the 3d mutant. Furthermore, mCherry-UNC93B1 itself was not detected in cortical ER domains by TIRF imaging. This indicates that the interactions between UNC93B1 and STIM1 are disrupted during the translocation process. UNC93B1 therefore exerts its chaperoning role in intracellular ER domains located at a distance >200 nm from the PM and does not reach the cortical and subcortical ER domains enriched in STIM1 oligomers. We postulate that in the absence of the chaperone, STIM1-YFP cannot rapidly switch into the extended conformation required for efficient trapping of Orai1 channels. Alternatively, UNC93B1 deficiency might impair the traffic or activity of an adapter protein to reduce STIM1–Orai1 interactions but not STIM1 recruitment (Fig. 7).

We could identify one mechanistic step impacted by the UNC93B1–STIM1 interactions, using STIM1 constructs bearing a single cysteine residue to study the formation of STIM1 dimers by crosslinking. These Cys mutants were engineered to establish the sequence of intradimer rearrangements occurring as STIM1 switches into an active conformation. We aimed to restrict the functional interactions by monitoring the formation of cross-linked dimers between Cys residues inserted within different interacting domains. Our results indicated that regardless of the position of the inserted Cys residue, the enforced expression of UNC93B1 facilitated the formation of dimer interaction within the STIM1 TM and proximal cytosolic helixes. This is consistent with UNC93B1 acting as a chaperone that assists the early steps of the STIM1 activation cascade to facilitate the initiation and extension of STIM1 dimers. We therefore propose that UNC93B1 is a chaperone that facilitates STIM1 early activation steps associated with ER Ca^2+^ depletion to enable the switch into an extended conformation required for the efficient trapping of Orai1 channels. After this step, STIM1 PM translocation and channel gating do not require UNC93B1, as STIM1 binds to negatively charged PM lipids *via* its polybasic tail and activates ORAI1 channels *via* its exposed CAD domain. In the absence of UNC93B1, unchaperoned STIM1 molecules can translocate when overexpressed but cannot interact efficiently with Orai1 channels. This defect is alleviated if sufficient time is provided to trap Orai1 channels, either *via* the delivery of additional STIM1 proteins or *via* the slow capture of sufficient Orai1 channels. By enhancing the translocation of extended STIM1 dimers, UNC93B1 chaperoning therefore maintains an optimal STIM1–Orai1 coupling during SOCE (Fig. 7).

## Experimental procedures

### Cell line, cell culture, and plasmids

HEK-293T cells were obtained from American Type Culture Collection (CRL-11268) and cultured in Dulbecco's modified Eagle's medium Glutamax (catalog no.: 31966-021; Gibco) with 10% fetal bovine serum (catalog no.: 10270106; Thermo Fisher Scientific) and 1% penicillin/streptomycin (catalog no.: 15140122; Gibco). HEK-293T cells lacking both endogenous STIM1 and STIM2 proteins, HEK-293T *STIM1/2*^*−/−*^, were obtained from Barbara Niemeyer (47). HEK-293T and HEK-293T *STIM1/2*^*−/−*^ cells were used to generate *UNC93B1* gene deletion using CRISPR–Cas9. Single-guided RNA with the sequence 5′-GCGGAGCGGAAACCTCATTGTGG-3′ was cloned into pSpCas9(BB)-2A-Puro-(pX459), purchased from Addgene (plasmid no.: 62988; http://n2t.net/addgene:62988; Research Resource Identifier: Addgene_62988), according to the published protocol (48). HEK-293T cells were transfected with the sgRNA-Cas9 plasmid using Lipofectamine 2000 (catalog no.: 11668019; Thermo Fisher Scientific), and transfection-positive cells were selected with puromycin (catalog no.: A1113803; Invitrogen) treatment. Sorted single clones were screened for UNC93B1 knockout by sequencing the genomic PCR product from the forward primer 5′-TGACTGTGTGCCCTACTCTCTG-3′ and the reverse primer 5′-GGCTGTACTTACCAAGGCGATA-3′ (Fig. S1*B*). The HEK-293T *STIM1*^*flip-in*^ cells were obtained from Trevor Shuttleworth (49). The mouse dendritic cell line 2114 was obtained from Isabelle Dunand-Sauthier (50) and cultured in Iscove's modified Dulbecco's medium (catalog no.: 31980; Gibco) with 10% fetal bovine serum (without lipopolysaccharide), 1% penicillin/streptomycin, and 50 μM 2-mercaptoethanol (catalog no.: 31350-010; Gibco). All cells were grown at 37 °C and 5% CO_2_.

The STIM1-YFP construct was a gift from Dr A.B. Parekh. The mCherry-STIM1 construct was a gift from Dr Richard S. Lewis. The mCherry-UNC93B1 and mCherry-UNC93B1 3d constructs were gifts from Dr Bénédicte Manoury. The UNC93B1-FLAG-MYC construct was obtained from Origene (catalog no.: RC210505). The Orai1-YFP construct was purchased from Addgene (catalog no.: 19756). The pcDNA3.1 plasmids carrying STIM1-A230C, STIM1-M241C, and STIM1-L251C were gifts from Dr Patrick Hogan. The STIM1-TM4-YFP was generated using Gibson Assembly (catalog no.: E2621S; NEB) of fragment 1, made by PCR of STIM1-YFP with the primers 5′-TCTGGTCTATCGTTACTCCAAGGAGCAC-3′ and 5′-CAATGATGGTGTCCTTGAGGTGATTATGG-3′, and fragment 2, made by PCR of TLR4-YFP (catalog no.: 13018; Addgene) with primers 5′-CCTCAAGGACACCATCATTGGTGTGTC G-3′ and 5′-TGGAGTAACGATAGACCAGAACTGCTACAAC-3’. The mCherry-STIM1 (1–152) was generated by introducing a STOP codon at amino acid 152, using site-directed mutagenesis with the following primers 5′-TCAGTATGAGTAGAGACCTTCCGG-3′ and 5′-GGCAGCTCCACATATGTG-3’. The mCherry-STIM1 (1–240) was generated with the same protocol with the following primers 5′-CCAAGGAGCATAGATGAAGAAGATGATG-3′ and 5′-AGTAACGGTTCTGGATATAG-3’.

### Immunoprecipitation and Western blot analysis

For immunoprecipitation, cells were lysed for endogenous protein interaction or were transfected prior to lysis for exogenous protein interaction. All cells were grown on a 10 cm dish to 90% confluency on the day of experiment. Cells were harvested in 500 μl of lysis buffer (0.5% NP-40, 50 mM Tris [pH 7.4], 150 mM NaCl, and 5 mM EDTA) with protease inhibitor (catalog no.: S8830; Sigma) for 30 min on ice and then pelleted at 800*g* for 15 min at 4 °C. An aliquot of the supernatant was collected for total lysate, and the rest was precleared for 30 min with rotation at 4 °C with 50 μl of 10% beads, protein A/G PLUS-Agarose (catalog no.: Sc-2003; LabForce), previously washed, and suspended in base buffer (50 mM Tris [pH 7.4], 150 mM NaCl, and 5 mM EDTA). The beads were pelleted with brief centrifugation, and the supernatant was collected and incubated overnight with rotation at 4 °C with 2 to 3 μl of the desired antibodies. The sample was then incubated with 100 μl of 10% beads the next day, for 4 h with rotation at 4 °C. The beads were pelleted and washed five times with base buffer and beads, and total lysates were suspended with NuPage LDS sample buffer (catalog no.: NP0007; Thermo Fisher Scientific) with 2% β-mercaptoethanol and subjected to Western blot analysis.

For Western blot analysis, 5 to 50 μg of proteins were loaded and separated on a 4 to 20% Mini-PROTEAN TGX Precast Protein Gel (catalog no.: 4561093; Bio-Rad). Proteins were then transferred to nitrocellulose membrane (catalog no.: 741290; Macherey–Nagel) followed by immunodetection. Rabbit anti-UNC93B1 (1:2000 dilution) were gifts from Dr Bénédicte Manoury, rabbit anti-STIM1 (1:2000 dilution; catalog no.: S6072; Sigma), rabbit anti-STIM2 (1:2000 dilution; catalog no.: 4917S; Cell Signaling), mouse anti-Orai1 (catalog no.: ab175040; Abcam), rabbit anti-calnexin (1:1000 dilution; catalog no.: ADI-SPA-865-D; Enzo), mouse anti-plasma membrane Ca^2+^-ATPase (1:2000 dilution; catalog no.: ab2825; Abcam), rabbit anti-Rab7 (1:1000 dilution; catalog no.: ab137029; Abcam), rabbit anti–lysosomal-associated membrane protein 1 (1:1000 dilution; catalog no.: ab24170; Abcam), rabbit anti-ORMDL3 (1:1000 dilution; catalog no.: ABN417; Sigma–Aldrich), mouse anti-FLAG (1:1000 dilution; catalog no.: F1804; Sigma), goat anti-GFP (1:1000 dilution; catalog no.: AB0020-20; SicGen), goat anti-GAPDH (1:1000 dilution; catalog no.: ab9483; Abcam), and mouse anti-γ-tubulin (1:1000 dilution; catalog no.: MA1-849; Thermo Fisher Scientific).

### PCR and quantitative PCR

Genomic DNA was isolated with GenElute Mammalian genomic DNA isolation kit (catalog no.: G1N70-1KT; Sigma), and PCR was carried out with GoTaq G2 Hot Start Green Master Mix (catalog no.: M7422; Promega). PCR product bands were detected following separation on a 1% agarose gel and isolated by cutting out the gel band and isolation with Monarch DNA gel extraction kit (catalog no.: T1020S; NEB).

Total RNA was isolated with Miniprep RNeasy Mini Kit (catalog no.: 74104; Qiagen), and first strand complementary DNA (cDNA) synthesis was carried out with Qscript cDNA synthesis Supermix (catalog no.: 95048-100; Quanta Biosciences). For the quantitative PCR, we used SensiFast SYBR Hi-ROX kit (catalog no.: BIO92005; Labgene), and the reaction was performed on StepOnePlus Real-Time PCR System (catalog no.: 4376600; Thermo Fisher Scientific). The following primers were used: 18s forward 5′-GGCCCTGTAATTGGAATGAGTC-3′, 18s reverse 5′-CCAAGATCCAACTACGAGCTT-3’; UNC93B1 forward 5′-CTGCTCACCTTCATCCTCTTT-3′, UNC93B1 reverse 5′-GTGCTGAGTCCAGTCTTGTT-3’; STIM1 forward 5′-CCTCTCTTGACTCGCCATAATC-3′, STIM1 reverse 5′-CTTGGAGTAACGGTTCTGGATATAG-3’.

### Subcellular fractionation

Cells were grown in 150 mm dishes to 90% confluency on the day of experiment, and three dishes were used per gradient separation. About 50% iodixanol working solution was made using Optiprep (catalog no.: D1556; Sigma–Aldrich) diluted with diluent buffer (0.25 M sucrose and 60 mM Tris [pH 7.4]). Dilutions (6%, 9%, 12%, 15%, 18%, 21%, 24%, and 27%) of iodixanol were prepared by diluting the working solution in homogenization buffer (0.25 M sucrose and 10 mM Tris [pH 7.4]). Gradients were prepared by layering 500 μl of each dilution in a polyallomer (13 × 51 mm) clear ultracentrifuge tube (catalog no.: 344057; Beckman). Gradients were prepared a day prior to cell harvest and stored overnight at 4 °C. Cells were washed twice with ice-cold PBS and scraped in 800 μl of ice-cold homogenization buffer with protease inhibitor. The homogenate was passed through a 14 micron clearance ball bearing homogenizer 25 times and centrifuged at 800*g* for 10 min at 4 °C. About 100 μl of the supernatant was kept as total, and 800 μl of the rest of the supernatant was layered on top of the previously prepared iodixanol gradient. The gradients were then centrifuged at 39,000 rpm for 6 h at 4 °C with a swinging bucket rotor (Beckman; SW 55 Ti) using the Beckman Ultracentrifuge Optima XPN100. About 12 fractions or 400 μl of each fraction were collected from the top of the gradient into 1.5 ml Eppendorf tubes. Proteins were precipitated by addition of three times the volume of 100% acetone and incubated at 4 °C overnight. The samples were then centrifuged at 13,000 rpm for 10 min at 4 °C to pellet the proteins, and the pellets were washed one time with 100% ethanol and recentrifuged. The resulting pellets were dissolved in sample buffer and prepared for Western blot analysis.

### *In vivo* crosslinking

*In vivo* crosslinking was adapted from a previous publication (51). Briefly, HEK*-STIM1/2*^*−/−*^*/UNC93B1*^*−/−*^ cells were grown and transfected with plasmids expressing STIM1 A230C, M241C, or L251C with pcDNA3 or pcDNA3 expressing UNC93B1-FLAG-MYC in a 6-well plate format. On the day of experiment, cells were treated or not with 0.5 μM Tg for 5 min and immediately incubated with 180 μM aldrithiol-4 (catalog no.: 143057; Sigma) for 20 min at 37 °C. Cells were then washed twice with cold PBS and lysed on ice for 15 min with 200 μl/well of TUNES buffer (100 mM Tris [pH 7.2], 6 M urea, 10 mM EDTA, 1% SDS, 0.4 M NaCl, and 10% glycerol) containing 50 mM *N*-ethylmaleimide (catalog no.: E1271; Sigma) and protease and phosphatase inhibitors. Cells were then scraped and sonicated with the Covaris S220 Focused-Ultrasonicator (Power 140 W, duty factor 10%, cycles per burst 200, 44 s) at the iGE3 Genomics platform in UNIGE. About 1% of bromophenol blue were added to the sonicated samples and prepared for Western blot analysis.

### Calcium imaging

Single-cell live imaging of cytosolic calcium was performed as previously described (52). Briefly, HEK-293T cells plated on 25 mm poly-l lysine-coated coverslips, grown to 80% confluency, were loaded with 4 μM Fura-2AM in modified Ringer's solution containing 2 mM CaCl_2_ for 25 min at room temperature, followed by washout and incubation with modified Ringer's solution containing 2 mM CaCl_2_ for 10 min at room temperature. Cells were imaged ratiometrically by 340/380 nm excitation and 510 ± 40 nm emission every 5 s. SOCE activity was induced by ER Ca^2+^ store depletion with 2 μM Tg (catalog no.: T9033/CAY10522; Sigma) or CPA (catalog no.: c1530; Sigma) in a Ca^2+^-free solution containing 1 mM EGTA, and Orai1-mediated Ca^2+^ entry was measured upon Ca^2+^addition. For induction of a second SOCE, CPA was washed out with modified Ringer's solution containing 2 mM CaCl_2_ and allowed time for ER Ca^2+^ refilling before the second round of CPA induction.

Fluorimetric recordings were performed in a 96-well microplate, coated with poly-l lysine, using the FDSS microcell plate imager (Hamamatsu) with 4 μM Fluo-8 (catalog no.: 210832; AAA Bioquest) as a calcium dye. Fluorescence was measured at 490 nm excitation and 525 nm emission, with automated washing and reagent additions.

### Duolink and TIRF microscopy

Duolink was performed with the Duolink *In Situ* Red Starter Kit Mouse/Rabbit (catalog no.: DUO92101-1KT; Sigma–Aldrich) according to the manufacturer's instructions. Briefly, HEK-293T cells were grown on 12 mm poly-l lysine-coated coverslips and fixed with 4% paraformaldehyde in PBS for 15 min at room temperature, washed with PBS, and permeabilized with 0.1% Triton X-100 in PBS for 10 min at room temperature. Cells were washed with PBS and incubated with 2% bovine serum albumin blocking solution for 1 h at room temperature. Primary antibodies (mouse anti-Orai1 and rabbit anti-STIM1) were incubated overnight at 4 °C. Cells were then washed with PBS, and proximal ligation assay probes were added, followed by hybridization, ligation, and amplification. Cells were mounted with nucleus staining (4′,6-diamidino-2-phenylindole) mounting media, and the STIM1–Orai1 interaction (*red*) was imaged with an LSM 700 Nikon confocal microscope.

STIM1–Orai1 colocalization and STIM1 movement at the TIRF plane were imaged with a Nikon Eclipse Ti microscope equipped with the Perfect Focus System (III) and the 100× oil CFI Apochromat TIRF Objective (numerical aperture of 1.49; Nikon Instruments). HEK-293T cells were plated on 25 mm poly-l lysine-coated coverslips and incubated in modified Ringer's solution containing 2 mM CaCl_2_. Images were taken at 30 s intervals following addition of 2 μM Tg, for 10 min at room temperature using the SET 488/10 and ZET 561/10 excitation filters (Chroma Technology Corp). Emission signals were recorded with a cooled EMCCD camera (iXon Ultra 897; Andor Technology Ltd).

### Image analysis and statistics

All images were analyzed using ImageJ software (Wayne Rasband and the National Institute of Mental Health). Colocalization was performed with a developed macro (GitHub; https://github.com/Carandoom/Coloc2ChannelsStackROI) using MATLAB. Duolink particle counting was performed as previously described (53).

## Data availability

All data described are located in this article.

## Supporting information

This article contains supporting information.

## Conflict of interest

The authors declare that they have no conflicts of interest with the contents of this article.
