# Supplementary material for: The mammalian trafficking chaperone protein UNC93B1 maintains the ER calcium sensor STIM1 in a dimeric state primed for translocation to the ER cortex
Source: J Biol Chem. 2022 Jan 20;298(3):101607. doi: 10.1016/j.jbc.2022.101607 (PMC8857484; doi:10.1016/j.jbc.2022.101607)
Supplement: Supplemental Figures S1–S8 [file mmc1.pdf]

The mammalian un-coordinated 93 homologue B1 maintains STIM1 in a dimeric state primed for translocation to cortical ER domains.

Wen-An Wang & Nicolas Demaurex*

Supporting Information

Figures and figure legends S1-S8

A

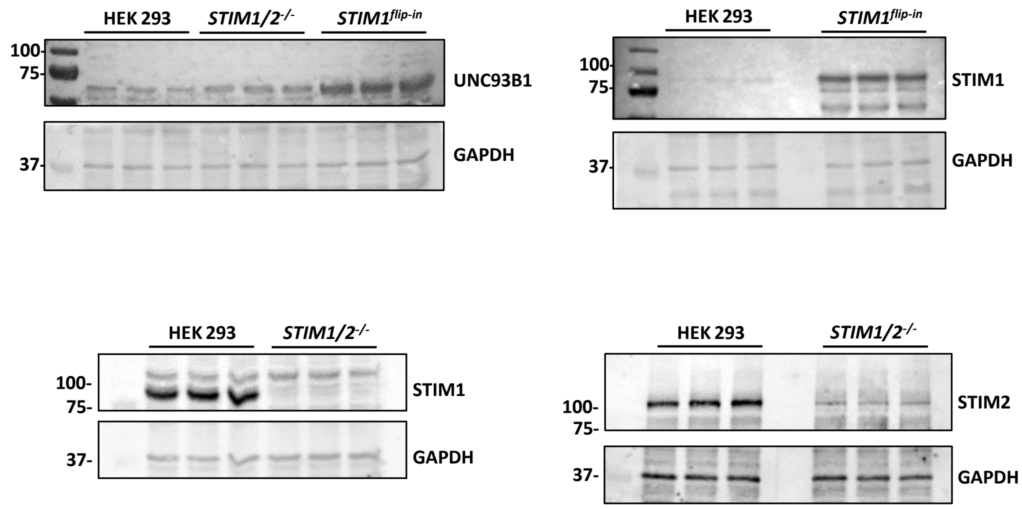

B Human UNC93B1 genome

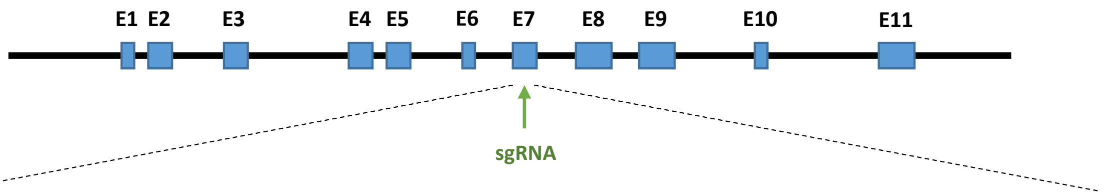

HEK 293 wild-type

----CTCAGCGGCTTCAACAAGACGGTTCTGCGGACGCTCCCGCGGAGCGGAAACCTCATTGTGGTGGAGAGCGTGCTCATGGCAGTGGCCTTCCTGGCCAT----

HEK 293 *UNC93B1*$^{-/-}$ (clone 52)

----CTCAGCGGCTTCAACAAGACGGTTCTGCGGACGCTCCCGCGGAGCGGAAACCTCA-------------GAGAGCGTGCTCATGGCAGTGGCCTTCCTGGCCAT----

HEK 293 *STIM*$^{-/-}$/*UNC93B1*$^{-/-}$ (clone 30)

----CTCAGCGGCTTCAACAAGACGGTTCTGCGGACGCTCCCGCGGAGCG----------------------GGTGGAGAGCGTGCTCATGGCAGTGGCCTTCCTGGCCAT----

HEK 293 *UNC93B1*$^{-/-}$ (clone 52)

Met E A E P P L Y P Met A G A A G P Q G D E D L L G V P D G P E A P L D E L V G A Y P N Y N E E E E E R R Y Y R R K R L G V L K N V L A A S A G
G Met L T Y G V Y L G L L Q Met Q L I L H Y D E T Y R E V K Y G N Met G L P D I D S K Met L Met G I N V T P I A A L L Y T P V L I R F F G T K
W Met Met F L A V G I Y A L F V S T N Y W E R Y Y T L V P S A V A L G Met A I V P L W A S Met G N Y I T R Met A Q K Y H E Y S H Y K E Q D G Q
G Met K Q R P P R G S H A P Y L L V F Q A I F Y S F F H L S F A C A Q L P Met I Y F L N H Y L Y D L N H T L Y N V Q S C G T N S H G I L S G F N K T E R
A H G S G L P G H A A G A G F V R S R L P A H G G D R S A Q R G L G Q H L P A A L Q A R A **Stop**

HEK 293 *STIM*$^{-/-}$/*UNC93B1*$^{-/-}$ (clone 30)

Met E A E P P L Y P Met A G A A G P Q G D E D L L G V P D G P E A P L D E L V G A Y P N Y N E E E E E R R Y Y R R K R L G V L K N V L A A S A G
G Met L T Y G V Y L G L L Q Met Q L I L H Y D E T Y R E V K Y G N Met G L P D I D S K Met L Met G I N V T P I A A L L Y T P V L I R F F G T K
W Met Met F L A V G I Y A L F V S T N Y W E R Y Y T L V P S A V A L G Met A I V P L W A S Met G N Y I T R Met A Q K Y H E Y S H Y K E Q D G Q
G Met K Q R P P R G S H A P Y L L V F Q A I F Y S F F H L S F A C A Q L P Met I Y F L N H Y L Y D L N H T L Y N V Q S C G T N S H G I L S G F N K T V L
R T L P R S G W R A C S W Q W P S W P C C W C W V C A E P L T G P R R R S I C A A W A G A T S S S C P S S T C V T T A C A T S C L S L S T A A S R C
S L P A L V S P W A **Met** A C A R W G W S G W L T S S W L T A W A P Q P P H S W A C W A C G C H A R C P W W P E Q G C T C C S P S S S F S G P L
C L G S C N T A G S S **Met** W Q L P F G V W A V P **Stop**

Figure S1

Figure S1

A. Western blot of protein lysates from WT, SKO and *STIM1*$^{flip\text{-}in}$ cells probed with αSTIM1, αSTIM2, αUNC93B1 and αGAPDH antibodies as indicated. B. Structure of the human *UNC93B1* gene with our sgRNA designed to target exon 7 (upper panel), sequencing results (middle panel) reveal base pair deletions within the sgRNA region translating in early stop codons within the amino acid sequence (in red, lower panel).

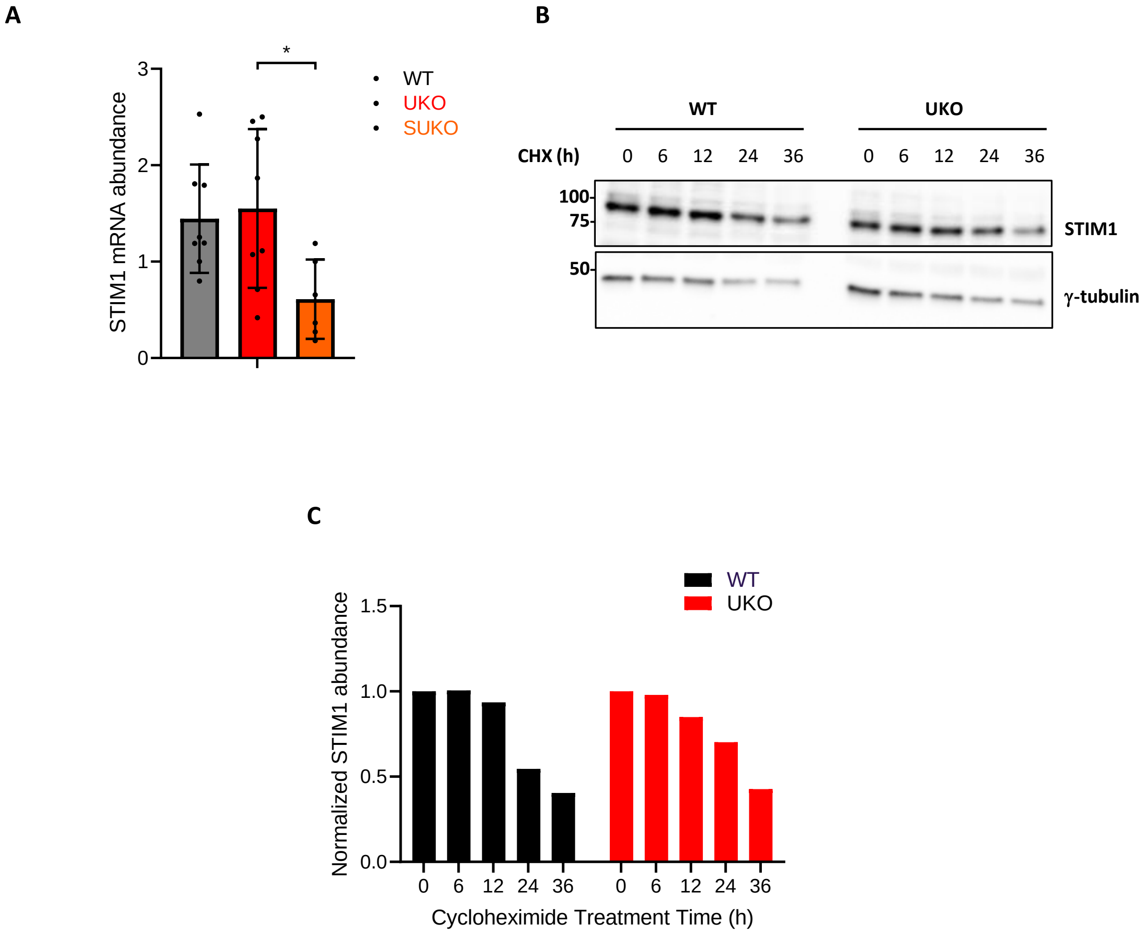

A
STIM1 mRNA abundance
0
1
2
3
*
WT
UKO
SUKO
B
WT
UKO
CHX (h)
0 6 12 24 36
0 6 12 24 36
100
75
50
STIM1
γ-tubulin
C
WT
UKO
Normalized STIM1 abundance
0.0
0.5
1.0
1.5
0 6 12 24 36
0 6 12 24 36
Cycloheximide Treatment Time (h)

Figure S2

Figure S2

A. STIM1 mRNA levels assessed by qPCR in WT, UKO and SUKO cells. N=4, *p<0.05, ordinary one-way ANOVA (F=4.241, P=0.0300). B. Western blot of protein lysates of WT and UKO cells treated with 30 μg/ml of cycloheximide (CHX) for 0, 6, 12, 24, 36 hours and probed with αSTIM1 and αγ-tubulin antibodies as indicated. C. Quantification of protein degradation in the western blots. Representative of 2 experiments.

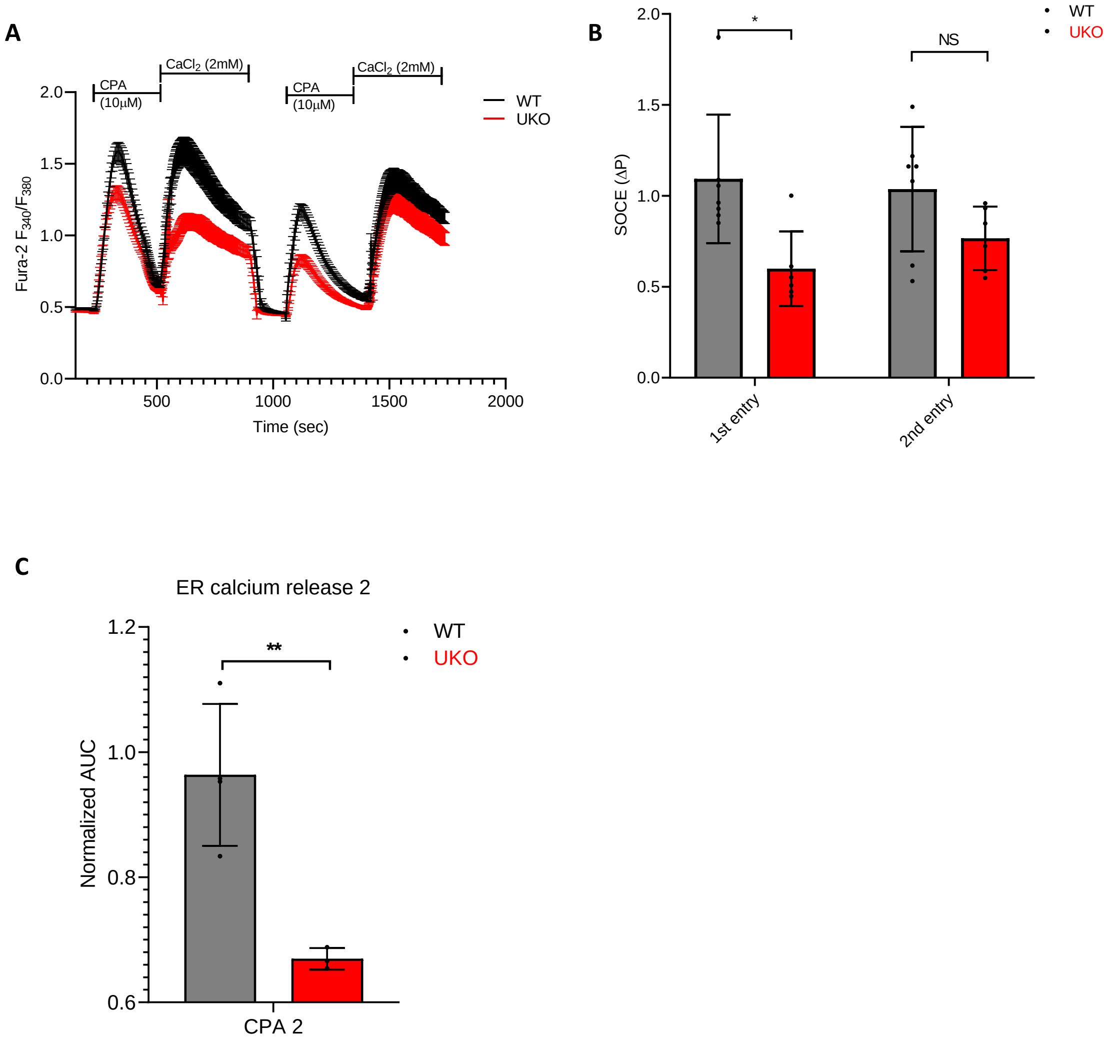
A
CPA
(10μM)
CaCl2 (2mM)
CPA
(10μM)
CaCl2 (2mM)
WT
UKO
Fura-2 F340/F380
2.0
1.5
1.0
0.5
0.0
500
1000
1500
2000
Time (sec)
B
2.0
1.5
1.0
0.5
0.0
SOCE (ΔP)
*
NS
WT
UKO
1st entry
2nd entry
C
ER calcium release 2
1.2
1.0
0.8
0.6
Normalized AUC
**
WT
UKO
CPA 2

Figure S3

Figure S3

A. Representative Fura-2AM recordings from WT and UKO cells during successive additions of 10 μM cyclopiazonic acid (CPA) in Ca^{2+}-free medium followed by readmission of 2 mM Ca^{2+}. B. Peak amplitude of the two successive SOCE responses. Data are mean±SD of 7 independent experiments. NS=non-significant; *p>0.05, two-way ANOVA (Interaction: SS=0.08050, DF=1, MS=0.08050, F(1,11)=2.132, P=0.1722). C. The ER Ca^{2+} mobilized after the second addition of CPA in Ca^{2+}-free medium as quantified with area under the curve (AUC). Data are mean±SD of 7 independent experiments. NS=non-significant; **p>0.01, unpaired t-test.

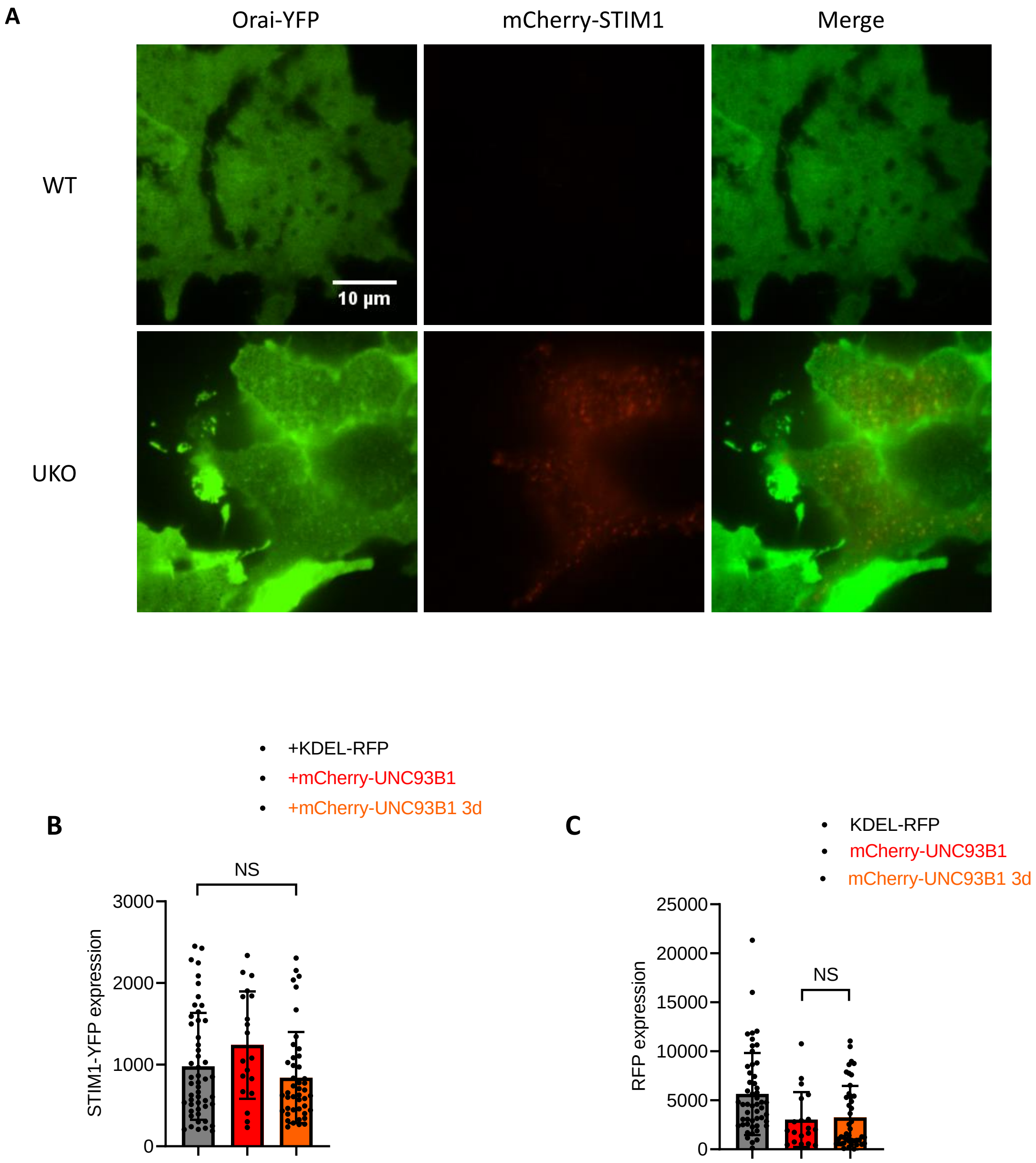
A
Orai-YFP
mCherry-STIM1
Merge
WT
10 µm
UKO
+KDEL-RFP
+mCherry-UNC93B1
+mCherry-UNC93B1 3d
B
NS
STIM1-YFP expression
3000
2000
1000
0
C
KDEL-RFP
mCherry-UNC93B1
mCherry-UNC93B1 3d
RFP expression
25000
20000
15000
10000
5000
0
NS

Figure S4

Figure S4

A. Corresponding representative TIRF images to Fig. 2C of WT and UKO cells expressing Orai1-YFP (green) and mCherry-STIM1 (red) at rest before TG treatment. B, C. Wide-field YFP (B) and RFP/mCherry (C) fluorescence levels of WT cells co-expressing YFP-STIM and RFP/mCherry constructs used for the TIRF experiments of Fig. 3. Data are mean±SD of 26-69 cells from 4 independent experiments. NS=non-significant, ordinary one-way ANOVA (B. F=2.779, P=0.664) (C. F=6.458, P=0.0022).

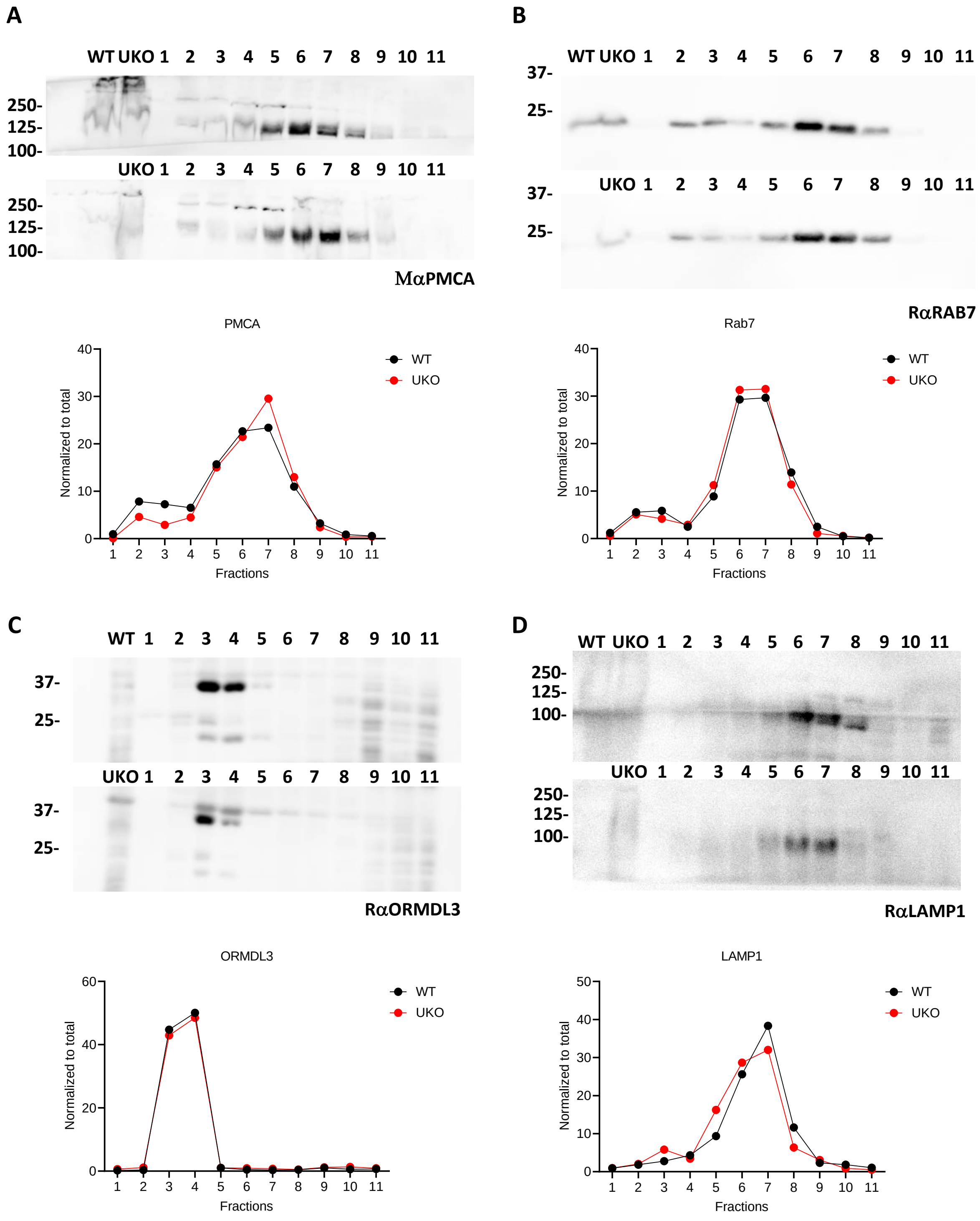
A
WT UKO 1 2 3 4 5 6 7 8 9 10 11
250-
125-
100-
UKO 1 2 3 4 5 6 7 8 9 10 11
MαPMCA
PMCA
Normalized to total
Fractions
WT
UKO
B
WT UKO 1 2 3 4 5 6 7 8 9 10 11
37-
25-
RαRAB7
Rab7
C
WT 1 2 3 4 5 6 7 8 9 10 11
RαORMDL3
ORMDL3
D
RαLAMP1
LAMP1

Figure S5

Figure S5

Immunoblot of protein lysates of WT (top) and UKO (bottom) cells, subjected to subcellular fractionation (1-11; light to heavy) through an iodixanol gradient (6%, 9%, 12%, 15%, 18%, 21%, 25%, and 27%). Blots were probed with αPMCA (A), αRab7 (B), αORMDL3 (C) and αLAMP1 (D) antibodies. Graphs show the quantification of the respective immunoreactivity through fractions 1 to 11. Representative of 2 biological experiments.

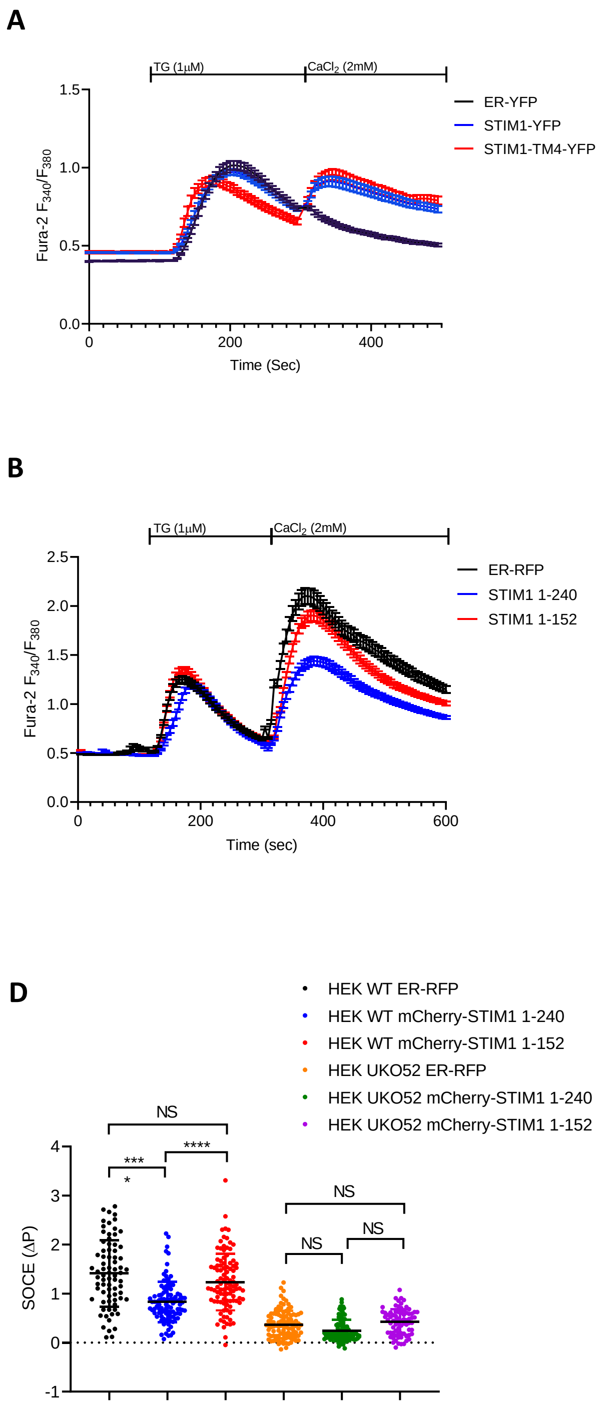
A
TG (1μM)
CaCl2 (2mM)
Fura-2 F340/F380
Time (Sec)
ER-YFP
STIM1-YFP
STIM1-TM4-YFP
B
TG (1μM)
CaCl2 (2mM)
Fura-2 F340/F380
Time (sec)
ER-RFP
STIM1 1-240
STIM1 1-152
C
TG (1μM)
CaCl2 (2mM)
Fura-2 F340/F380
Time (sec)
ER-RFP
STIM1 1-240
STIM1 1-152
D
HEK WT ER-RFP
HEK WT mCherry-STIM1 1-240
HEK WT mCherry-STIM1 1-152
HEK UKO52 ER-RFP
HEK UKO52 mCherry-STIM1 1-240
HEK UKO52 mCherry-STIM1 1-152
SOCE (ΔP)
NS
***
*
****
NS
NS
NS

Figure S6

Figure S6

Functional SOCE assessment of the STIM1-TM4, $STIM1^{1-240}$, and $STIM1^{1-152}$ truncation constructs. A. Averaged Fura-2 responses evoked by Tg and subsequent Ca^{2+} readmission to SKO cells expressing ER-YFP, STIM1-YFP or STIM1-TM4-YFP. B, C. Representative averaged Fura-2 responses evoked by Tg and subsequent Ca^{2+} readmission to WT (B) and UKO cells (C) expressing ER-RFP, $STIM1^{1-240}$ or $STIM1^{1-152}$. D. Peak amplitude of the SOCE responses in B-C. Data are mean±SD of 75-100 cells from 3 independent experiments. NS=non-significant, ****p>0.001, ordinary one-way ANOVA (F=112.6, P<0.0001).

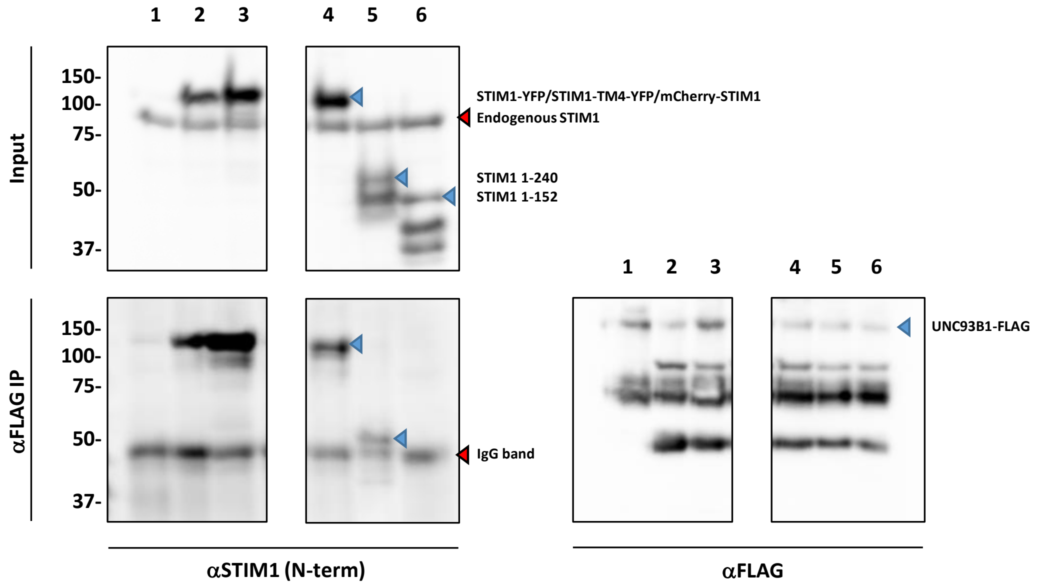
1 2 3
4 5 6
Input
αFLAG IP
150-
100-
75-
50-
37-
STIM1-YFP/STIM1-TM4-YFP/mCherry-STIM1
Endogenous STIM1
STIM1 1-240
STIM1 1-152
IgG band
UNC93B1-FLAG
αSTIM1 (N-term)
αFLAG

Figure S7

Figure S7

Immunoblot of input and immunoprecipitated (IP) protein lysates from WT cells expressing UNC93B1-FLAG with KDEL-GFP (1), STIM1-YFP (2), $STIM1^{TM}$-YFP (3), mCherry-STIM1 (4), mCherry-$STIM1^{1-240}$ (5), or mCherry-$STIM1^{1-152}$ (6). UNC93B1 was immunoprecipitated with αFLAG antibody and the blots were probed with αSTIM1 (N-term) and αFLAG antibodies. The blue arrows indicate the locations of the expected products and the red arrows additional reactivities. Representative of 2 biological experiments.

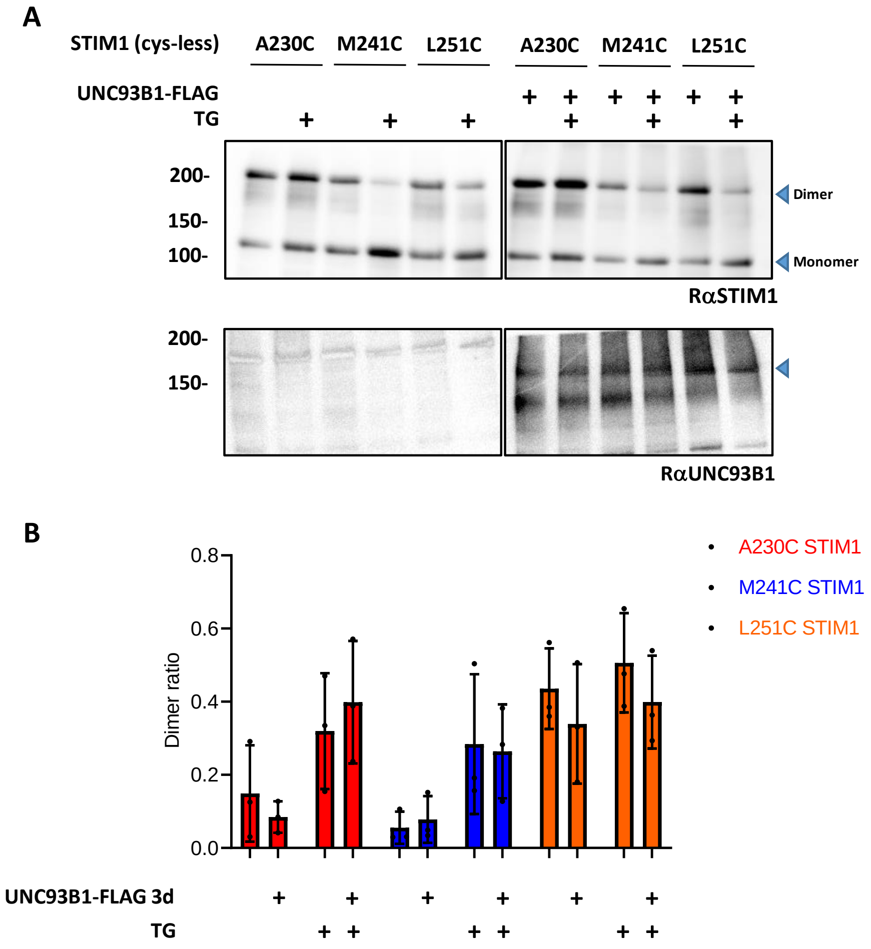
A
STIM1 (cys-less)
A230C
M241C
L251C
A230C
M241C
L251C
UNC93B1-FLAG
TG
200-
150-
100-
Dimer
Monomer
RαSTIM1
200-
150-
RαUNC93B1
B
Dimer ratio
0.8
0.6
0.4
0.2
0.0
A230C STIM1
M241C STIM1
L251C STIM1
UNC93B1-FLAG 3d
TG

Figure S8

Figure S8

A. Immunoblot analysis of protein lysates from SUKO cells expressing the single cysteine STIM1 constructs with or without mCherry-UNC93B1-3d and treated or not with Tg (0.5 μM for 5 min). Cells were subjected to *in-vivo* crosslinking with aldrithiol-4 and to electrophoresis under non-reducing conditions. Blots were probed with αSTIM1 and αUNC93B1 antibodies. Representative of 3 independent experiments. B. Quantification of the STIM1 dimer ratio from the *in-vivo* crosslink immunoblots. Data are mean±SD of 3 experiments. Non-significant where there is no indication, unpaired t-test.
